# Method for Pulmonary Administration Using Negative Pressure Generated by Inspiration in Mice

**DOI:** 10.3390/pharmaceutics12030200

**Published:** 2020-02-25

**Authors:** Yuki Oiso, Tomomi Akita, Daiki Kato, Chikamasa Yamashita

**Affiliations:** 1 Department of Pharmaceutics and Drug Delivery, Faculty of Pharmaceutical Sciences, 2641 Yamazaki, Noda, Chiba 278-8510, Japan; 3b14622@ed.tus.ac.jp (Y.O.); akitat@rs.tus.ac.jp (T.A.); 3b17616@ed.tus.ac.jp (D.K.); 2 Fusion of Regenerative Medicine with DDS, Research Institute for Science and Technology, Tokyo University of Science, 2641 Yamazaki, Noda, Chiba 278-8510, Japan

**Keywords:** pulmonary administration, chronic obstructive pulmonary disease, all-trans-retinoic acid

## Abstract

When developing inhaled medicines for respiratory diseases, such as chronic obstructive pulmonary disease, drugs need to be administered by pulmonary delivery to animals in non-clinical tests. Common methods require application of pressure during administration, and it may cause lung injury, so we focused on the inhalation of liquid medicines by mice themselves. This study aimed to evaluate a negative pressure method of pulmonary administration in mice by self-inhalation. First, to confirm the accuracy of delivery of liquid medicines into lungs and the potential for lung injury, Institute of Cancer Research (ICR) mice received methylene blue tetrahydrate or saline by the negative pressure method. We assessed drug distribution and usefulness of this method by administering porcine pancreatic elastase and all-trans-retinoic acid (ATRA) to mice. Consequently, we confirmed good distribution of the dye and no injury such as disruption of blood flow or destruction of alveoli in lungs of mice. Following production of the murine emphysema model, the mean linear intercept (Lm) was calculated as 78 ± 4 μm. Moreover, a significant therapeutic effect of administration of the ATRA was confirmed. These results suggest that this negative pressure method of administration may be useful for pulmonary administration in non-clinical tests.

## 1. Introduction

Chronic obstructive pulmonary disease (COPD) is a refractory inflammatory disease of lungs caused by inhalation of harmful substances, and it leads to airway obstruction and destruction of alveolar structures. Long-acting muscarinic antagonists, inhaled steroids, and long-acting β2-antagonists are used as symptomatic treatments [1], and these are mainly inhaled drug therapies [2,3].

When developing drugs for inhalation, it is also necessary to administer drugs via pulmonary delivery to animals. Pulmonary administration has attracted attention as an efficient method of delivering drugs directly into the blood circulation, and that scarcely affects drug metabolism. Moreover, pulmonary administration is an excellent method of delivering drugs directly to the lungs. Enna and Schanker reported a forced method of pulmonary administration in rats by cutting the trachea and injecting a solution [4]. Furthermore, Okamoto et al. developed a method of pulmonary administration for powder formulations using an apparatus with two 3-way stopcocks [5]. These methods require invasive cutting of the trachea of animals, which can cause an inflammatory reaction or bleeding, and thus, frequent administrations are difficult. However, various other methods of pulmonary administration to small animals that do not require tracheal cutting have been reported [6,7,8]. In these methods, intratracheal instillation or aerosol administration was used. As these methods do not involve a tracheostomy, they are considered to be non-invasive administration methods that do not cause damage, such as bleeding. However, regardless of whether the trachea is incised, in these methods, it is necessary for the experimenter’s hand to push a plunger to inject a drug solution, and to use compressed air (positive pressure) at the time of administration. If the injection speed is high, that is, the pressure applied at the time of administration is strong, the drug solution is likely to be evenly distributed in the lung [9]. Contrary to the benefit of high-speed injection, the pressure generated by the administration may cause lung damage, and differences in drug distribution between different experimenters, which can lead to incorrect evaluation of results. As the experimenter-associated issues of air pressure may affect administration of liquid medicines, we decided to focus on inhalation of liquid medicine by mice themselves. Pulmonary administration of a drug solution by respiration to mice may reduce the possibility of injury, and is a more suitable administration method for assessing pharmacokinetic and pharmacological effects.

To evaluate the therapeutic effect on emphysema in COPD, porcine pancreatic elastase (PPE) was administered intratracheally to create elastase-induced animal models of emphysema [10,11,12]. Further, all-trans-retinoic acid (ATRA) has been shown to have an alveolar repair effect in an elastase-induced rat model of emphysema [10]. In the present study, we aimed to evaluate a negative pressure method of pulmonary administration to mice by creating a pathological model of emphysema and assessing the therapeutic effects of ATRA using this method. We also used a staining solution to compare the negative pressure method to the positive pressure method in terms of drug distribution and lung injury. Finally, to demonstrate that the negative pressure method is useful for non-clinical testing, we confirmed that the negative pressure method of administration could produce a mouse model of elastase-induced emphysema and show the therapeutic effects of ATRA on collapsed alveoli in mice with COPD.

## 2. Materials and Methods

### 2.1. Animals and Materials

Male Institute of Cancer Research (ICR) mice were purchased from Sankyo Labo Service Corporation, Inc. (Tokyo, Japan). Animals were housed in a temperature-controlled (24 ± 1 °C) facility maintained on a 12-h light:12-h dark cycle with standard food available ad libitum for 1 week. All animal care and use procedures were approved by the Tokyo University of Science Ethics Committee (approval numbers: Y14052, Y15042, Y16044).

Isoflurane used for anesthetizing the mice, PPE, ATRA, dimethyl sulfoxide (DMSO), Tween80, methylene blue tetrahydrate, Mayer’s hematoxylin solution, 0.5% eosin Y, ethanol solution, absolute ethanol, and saline were purchased from Wako Pure Chemical Industries (Osaka, Japan).

### 2.2. Methods of Pulmonary Administration

**Figure 1:**
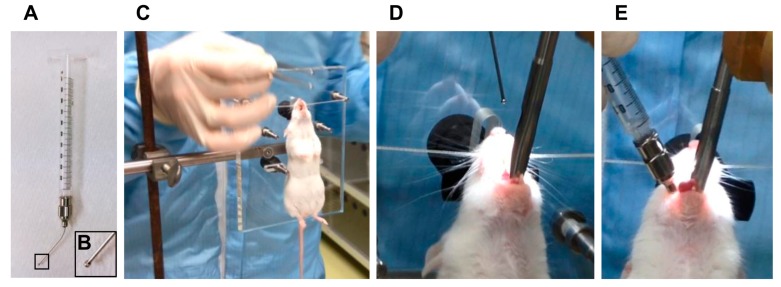
Steps for pulmonary administration to mice using the self-inhalation method. (**A**) Set-up of the administration syringe. (**B**) Enlarged view of the tip of the oral sonde. (**C**) Front teeth of the anesthetized mouse are retained at approximately 90° in the retaining position. (**D**) Visualization of the trachea using a small animal laryngoscope. (**E**) A feeding needle attached to a syringe is inserted into the trachea of the mouse, and the test agent is self-inhaled by the mouse without using the syringe plunger.

Pulmonary administration using the negative pressure method was carried out with a stainless steel oral sonde (Figure 1A) for oral administration (No. KN-348, for mice; Natsume Seisakusho, Tokyo, Japan), which is equivalent in diameter to the mouse airway. The length of the oral sonde is 2.5 cm, and the thickness of the sonde is 20–22 G, which matches the diameter of the mouse airway. Using the Mouse Intubation Platform—Model MIP (Penn–Century, Inc., Wyndmoor, PA, USA) as a mouse retainer for pulmonary administration, the front teeth of the mouse were retained at approximately 90° in the retaining position to facilitate tracheal access of the oral sonde (Figure 1B). The airway was confirmed with the Small Animal Laryngoscope—Model LS-2 (Penn–Century, Inc.) as a tracheal endoscope for the mouse (Figure 1C), the oral sonde was inserted into the airway, and the drug or dye solution was administered with negative pressure of inhalation generated by the mouse (Figure 1D). In Figure 1D, because of adherence of the oral sonde to the trachea, the drug is administered with one inhalation due to the negative pressure generated by the inhalation (Video S1). Sometimes, on exhalation, a portion of the drug may return to the syringe, but retaining the oral sonde in the trachea for a while can help avoid that phenomenon at administration.

Pulmonary administration with positive pressure was conducted using a MicroSprayer^®^ (Penn–Century, Inc.) in the usual manner. The MicroSprayer^®^ was filled with a drug or dye solution according to the manufacturer’s protocol. The mouse was retained, and the MicroSprayer^®^ was inserted into the airway as in the negative pressure method. Once the MicroSprayer^®^ was inserted into the airway, the plunger was strongly pushed to inject the drug or dye solution.

### 2.3. Effect of Pulmonary Administration on Lung Damage and Drug Distribution

We administered 50 µL saline to six-week-old male ICR mice using the negative pressure method or the positive pressure method. Immediately after the administration, the mice were sacrificed by excessive inhalation of isoflurane. A 3% solution of methylene blue tetrahydrate dissolved in 50 µL saline was administered to six-week-old male ICR mice using the negative pressure method or the positive pressure method. After 1 min of administration, the mice were sacrificed by excessive inhalation of isoflurane.

### 2.4. Mouse Model of Elastase-Induced COPD

Six-week-old male ICR mice were anesthetized with isoflurane, and a solution of PPE (0 or 7.5 units/50 µL of saline) was administered to lungs using the negative pressure method. The mice were sacrificed 2 weeks after the administration of PPE.

### 2.5. Administration of ATRA Using the Negative Pressure Method

Six-week-old male ICR mice were anesthetized with isoflurane, and a solution of PPE (7.5 units/50 µL of saline) was administered to lungs using the negative pressure method. After 1 week of PPE administration, 2.5 mg/kg ATRA was administered to lungs of the elastase-induced emphysema model mice twice a week for 2 weeks. ATRA was dissolved in 50 µL saline with 10% DMSO and 0.01% Tween80. Mice (*n* = 6) were sacrificed 2 weeks after the start of ATRA administration.

### 2.6. Computed Tomography

Mice were anesthetized with isoflurane and placed in the chamber of a Latheta LCT-200 computed tomography (CT) scanner for small animals (Aloka, Tokyo, Japan). The CT scanner was calibrated according to the manufacturer’s recommendations. CT scans were performed at 192-µm intervals (100 slices) with respiratory-gated image acquisition. The images captured between the apex and the base of the lung were used for quantitative assessment with Latheta software v. 3.2. The region of interest (ROI) was set at −871 to −610 Hounsfield units (HU) for analysis of the low attenuation area (LAA), which represents the area of lung damage [13]. Lung CT images were used to create 3D models by Amira, a system for 3D visualization and analysis (Visualization Sciences Group, Burlington, MA, USA). LAA sites in the emphysema area were visualized in red.

### 2.7. Pulmonary Histology

Tissue sections were stained with hematoxylin and eosin. The mean linear intercept (Lm), an indicator of air space size, was calculated for each mouse from 24 randomly selected fields.

### 2.8. Statistical Analysis

For each measured parameter, the values obtained from individual samples were averaged, and the standard error (S.E.) was calculated. Data were compared using the unpaired Student’s *t*-test. A 5% probability was considered significant.

## 3. Results

### 3.1. Comparison of the Negative and Positive Pressure Administration Methods

**Figure 2:**
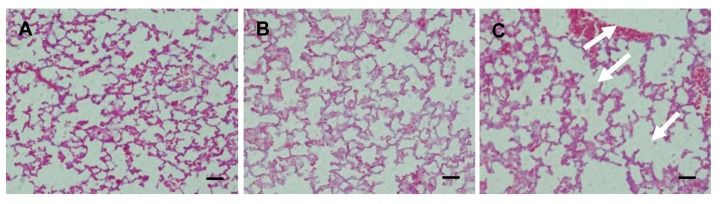
Lung sections from each administration method. Representative microscopic views of alveoli in (**A**) a non-saline-treated mouse, (**B**) a saline-treated mouse (negative pressure method), and (**C**) a saline-treated mouse (MicroSprayer^®^). After 1 min of saline administration, the lung was removed. Lung sections were stained with hematoxylin and eosin. The injuries that appeared are highlighted with white arrows. Scale bar = 40 μm.

**Figure 3:**
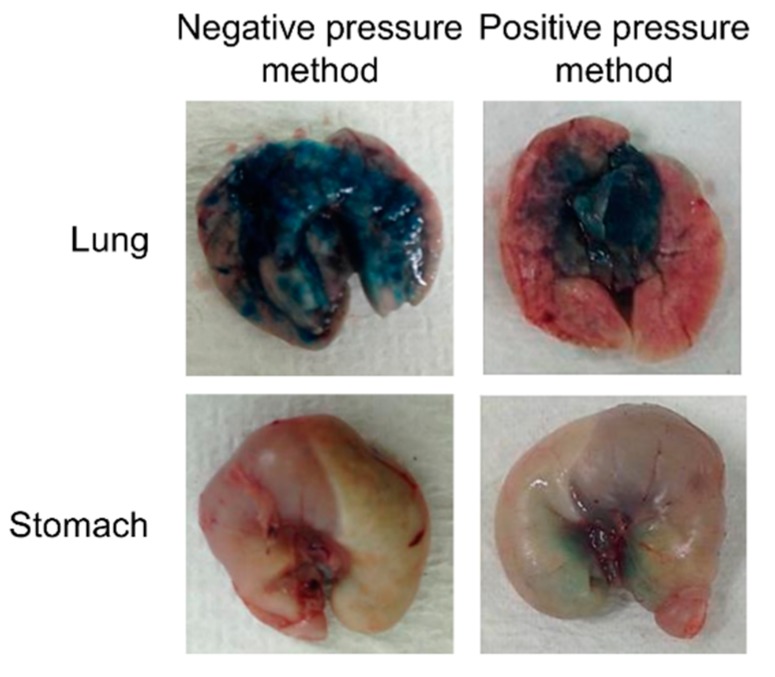
Macroscopic features of lungs and stomachs after pulmonary administration using the negative pressure method and the positive pressure method.

We evaluated whether the negative pressure method caused minimal damage to lung tissue by observing alveoli microscopically after 50 µL saline was administered to mice using the negative pressure method or the positive pressure method. In the evaluation of lung tissue injury caused by the administration method, non-treatment with saline resulted in clear alveoli (Figure 2A), and the negative pressure method did not destroy any of the alveoli (Figure 2B). However, collapsed alveoli were observed in some of the mice treated with positive pressure (Figure 2C). To evaluate the effects of differences in the administration method on drug distribution in the lung, a dye solution was administered using the negative pressure method or the positive pressure method. We used the dye solution to visually evaluate whether the negative pressure method could deliver liquid medicines to a lung more uniformly than the positive pressure method. The appearance of the lung and the stomach was observed. The negative pressure method delivered the dye solution to the lung uniformly. However, with the positive pressure method, the dye solution was present only in the center of the lung, and did not reach the periphery of the lung (Figure 3).

### 3.2. Usefulness of the Method with Negative Pressure in Inducing Emphysema in a Mouse Lung

**Figure 4:**
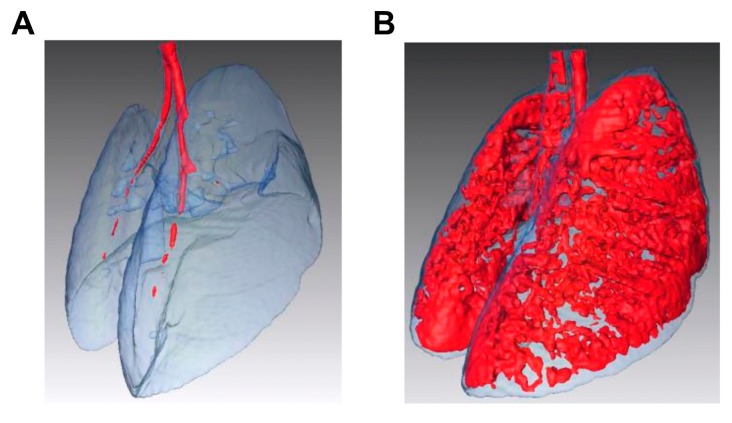
Micro-computed tomography (CT) assessment of elastase-induced chronic obstructive pulmonary disease model mice treated with porcine pancreatic elastase (PPE) using the negative pressure method. Three-dimensional images were obtained by integrating CT images of the mice treated with (**A**) 0 units of PPE (i.e., with saline alone) or (**B**) with 7.5 units of PPE. The low attenuation area (from −871 to −610 Hounsfield units (HU)) is colored in red, and the whole lung field (from −1000 to −200 HU) is colored in blue.

To confirm the usefulness of the negative pressure method, we examined whether emphysema could be induced in a mouse lung. Elastase-induced COPD model mice that were treated with 0 (saline only) or 7.5 units of PPE were evaluated by CT scanning. The LAA area, which is an emphysema lesion, shown in red in Figure 4 was observed uniformly in the whole lung.

### 3.3. Effect of Administration of ATRA Using the Negative Pressure Method on Pulmonary Physiology and Histology

**Figure 5:**
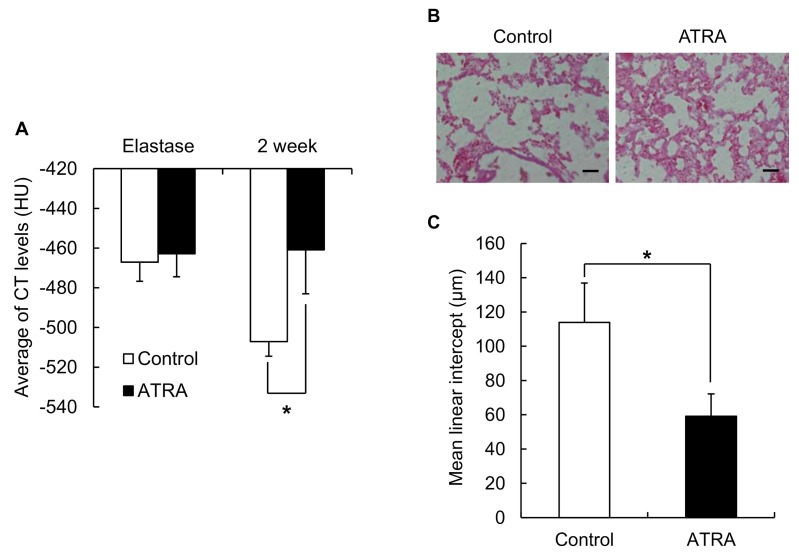
All-trans-retinoic acid (ATRA)-abrogated alveolar wall distance in elastase-induced chronic obstructive pulmonary disease model mice. (**A**) The average CT value, (**B**) lung sections (hematoxylin and eosin stain), and (**C**) the average air space in the mice treated with 2.5 mg/kg ATRA and in the control group at 2 weeks after the administration of ATRA. Scale bar = 40 μM. Data represent the mean ± S.E. * *p* < 0.05 (Student’s *t*-test) (*n* = 8 for the control group and *n* = 6 for ATRA in the treatment group, *p* = 0.047).

Finally, we investigated the administration of a drug using the negative pressure method to determine the actual therapeutic effects on pulmonary physiology and histology. We used ATRA as an alveolar regeneration drug. In the evaluation of pulmonary physiology based on our previous study, when the average CT value dropped below −450 HU, we identified that the emphysema was induced. The average CT value at 2 weeks was −507 ± 14 HU in the control group, and −461 ± 13 HU in the Am80-treated mice (Figure 5A). After the ATRA treatment, the average CT value was significantly reduced compared with the control group. In the evaluation of pulmonary histology, the Lm values were 114 ± 23 μm in the control group and 59 ± 13 μm in the ATRA-treated group (Figure 5C). These results indicate that it is possible for the administration of liquid medicines using the negative pressure method to have an adequate therapeutic effect on pulmonary physiology and histology.

## 4. Discussion

In this study, we proposed a negative pressure method for pulmonary administration and showed its usefulness for non-clinical testing on mice from several standpoints: delivery of a drug to a lung only, administration of a drug with low injury, and demonstration of effects by sufficient distribution of the drug. The most distinctive feature of our negative pressure method is that the administration is completed spontaneously by inhalation by the mouse. We do not need to cut the mouse’s trachea or push a syringe plunger during administration. In the evaluation of lung damage, our negative pressure method caused no bleeding, and only minimal damage to the alveoli (Figure 2). Drug administration with positive pressure was used and evaluated, too [14,15,16]. However, total lung volume and the volume of alveolar air in a mouse are about 0.9 to 1.9 cm^3^ and 0.5 to 1.0 cm^3^, respectively [17]. Therefore, it is possible to injure the lung by high air pressure during administration with positive pressure, because the lung is a nearly closed system while the tube is inserted. These results suggest that administration using the negative pressure method can help deliver a drug solution to the alveoli with very little injury. Furthermore, the evaluation using a dye showed that the negative pressure method can deliver a drug specifically to a lung, and uniformly within it (Figure 3). This result is different from a previous report, in which a drug solution was distributed in both the lungs and the stomach using the positive pressure administration method [18]. In our method, the drug solution was administered when the oral sonde was inserted in the airway of a mouse, because this method mainly depends on inhalation by the mice, and not on positive pressure. Therefore, the drug solution was delivered to the lung more efficiently and with greater certainty than when using positive pressure. Recently, many researchers have reported methods or procedures of non-invasive intratracheal intubation [19,20,21]. There are some points of resemblance in our method, although we use the Venturi effect. In the administration methods of these previous reports, unlike in our method, the drug solution was filled in a syringe or a sonde filled with the drug solution was replaced after inserting the sonde. In addition, instead of the drug being administered all at once, pulmonary administration to mice was performed with several inhalation operations, because there is a gap between the inserted sonde and the mouse trachea, and insufficient adhesion between the sonde and the trachea, so sufficient negative pressure is not applied, and not much of a Venturi effect to increase the administration speed of the drug solution can be expected. If sufficient negative pressure is generated, an efficient Venturi effect can be obtained, and the spray speed of the drug solution can be increased. As a result, effective aerosolization and distribution in the lung can be expected. Therefore, in our pulmonary administration method, we used a sonde with a round tip and small inner diameter. The trachea tightly adheres to this sonde; this prevents creating a gap and damaging the trachea. Thus, the administration speed of the drug solution is increased, and an effective Venturi effect is obtained. Another advantage of our administration method is that, unlike in the previous papers, the inner diameter of the sonde is small, so that a sonde filled with a drug solution can be inserted directly without dripping the drug solution from the sonde, and the drug solution can be administered in a single step. However, this method is not applicable to the administration of larger drug volumes or highly viscous solutions, because it can lead to asphyxiation of the mouse.

To determine the effects of a drug based on its sufficient distribution, we evaluated whether PPE delivered using our method could induce emphysema in mice, and would show a therapeutic effect. In the induced emphysema model, CT imaging was evaluated. As shown in Figure 4, the LAA region showed emphysema was observed throughout the lung. This suggested that elastase, which causes emphysema, was distributed throughout the lung by our pulmonary administration method. To evaluate pharmacological effects, the negative pressure method needs to show therapeutic effects of its use in an animal model. To evaluate the therapeutic effect, we used ATRA, which is reported to have an alveolar-repairing effect [10]. As a result, Lm decreased following the pulmonary administration of ATRA using the negative pressure method (Figure 5). This suggested that the negative pressure method delivered ATRA to the alveoli, which resulted in a therapeutic effect. One concern is that mice with decreased respiratory function, such as emphysema model mice, cannot sufficiently inhale liquid medicines. However, in this study, we could achieve a significant therapeutic effect in the elastase-induced emphysema model mice using the negative pressure method pulmonary administration; this indicates that this method can likely be applied to pharmacological testing using model mice. In this study, we have proposed a method for liquid administration to mice. As it is difficult to administer powder to mice using this method, it would be desirable to develop a device and a method to effectively administer powder in the future.

## 5. Conclusions

A negative pressure method of pulmonary administration controlled by the respiration of mice was evaluated and found to cause no harm to lung tissue, because it delivers the drug moderately by the inhalation of mice. In addition, this study revealed that the negative pressure method showed a significant therapeutic effect on pulmonary histology when a drug that can regenerate collapsed alveoli was administered in a mouse model of elastase-induced emphysema. These results suggest that the negative pressure method has the potential to become a useful method for pulmonary administration in non-clinical testing.

## Supplementary Materials

The following are available online at https://www.mdpi.com/1999-4923/12/3/200/s1, Video S1: Supplementary video for pulmonary administration.
